# Draft genome sequence of furan-degrading *Rhodococcus erythropolis* strain FUR100

**DOI:** 10.1128/mra.01127-23

**Published:** 2024-01-24

**Authors:** Steffen Helbich, Christine Woiski, Daniel Dobslaw, Thomas Gerl, Yevhen Vainshtein, Kai Sohn, Karl-Heinrich Engesser

**Affiliations:** 1 Department of Biological Waste Air Purification, University of Stuttgart, Institute for Sanitary Engineering, Water Quality and Solid Waste Management, Stuttgart, Germany; 2 Fraunhofer Institute for Interfacial Engineering and Biotechnology, Stuttgart, Germany; University of Southern California, USA

**Keywords:** furan, *Rhodococcus erythropolis*, microbial degradation

## Abstract

*Rhodococcus erythropolis* FUR100 was isolated from a mixture of soil and activated sludge. It can use furan as a sole source of carbon and energy. Its draft genome sequence may provide insight into the genetics of furan catabolism.

## ANNOUNCEMENT

Furan is an aromatic five-membered heterocyclic compound containing one oxygen atom. It is a precursor used in the synthesis of fine chemicals (1). In rat and human liver, furan is transformed by P450 monooxygenases to the carcinogenic cis-2-butene-1,4-dial, possibly via the unstable intermediate 2,3-epoxy-2,3-dihydrofuran (2–4). Bacterial aerobic metabolism of furan derivatives furfural, 2-furoic acid, 5-hydroxy-methylfurfural, and dibenzo[b,d]furan is well researched (5–7) but not of the heterocycle itself. *Rhodococcus erythropolis* FUR100 can use furan as a sole source of carbon and energy. The strain was obtained by enrichment in 100 mL liquid mineral salt medium (8), inoculated with a mixture of 10 g soil sample and 10 mL activated sludge from a wastewater treatment plant (Stuttgart-Büsnau, Germany, 48.75191 N 9.08968 E), supplied with 5 mM furan, and incubated at 30°C, 250 rpm, in a baffled flask (500 mL), sealed gas tight with a polytetrafluoroethylene septum. After 7 days, 1 mL of suspension was transferred to fresh medium supplied with furan and incubated until a visible increase in turbidity was observed. This step was repeated a second time. A dilution of a 1 mL sample was plated on mineral salts medium plates and incubated at 30°C in a desiccator supplied with a 0.001% furan atmosphere. Single colonies were picked and streaked onto fresh plates to obtain pure cultures. These pure cultures were tested again for growth in liquid culture to exclude the use of agar as a carbon source. One of the isolated strains, FUR100, was further examined.

DNA was obtained from a fresh liquid culture (same conditions as isolation procedure). Cells were harvested after 4 days, washed twice with PBS (4°C, 10,000 × *g*, 10 min), and resuspended in 5 mL PBS. Cell pellets were created by pipetting droplets (~100 µL) directly into liquid nitrogen and disrupted using a Mixer Mill MM 200 (Retsch). DNA was isolated with the QIAamp DNA Mini Kit (Qiagen) according to the manufacturer’s protocol. The sequencing library was prepared from 50 ng of input material using the Nextera DNA Library Preparation Kit (Illumina) according to the manufacturer’s protocol and sequenced on an Illumina HiSeq2500 platform in paired-end mode for 150 cycles. A sequencing depth of 5,866,912 reads was generated. Sample integrity and sequencing library quality were ensured by using a Fragment Analyzer (Agilent). Raw sequencing reads were demultiplexed with the bcl2fastq software (Illumina, 2015). Default parameters were used for all software unless otherwise specified. Adapter trimming and low-quality read removal were performed using BBDuk (BBMap v34.41 http://sourceforge.net/projects/bbmap/) with the parameters: trimpolyg = 10 hdist = 1 mink = 12 maxns = 10 minlen = 50 trimq = 20 qtrim = t ktrim = r k = 28. The quality of the sequencing was controlled with FastQC (http://www.bioinformatics.babraham.ac.uk/projects/fastqc/). The genome was *de novo* assembled using ABySS 2.0.2 (9), followed by an evaluation with Quast v5.0.2 (10). The genome was annotated by NCBI using PGAP (11). It has a size of 7,667,056 bp with a GC content of 62.5% and a coverage of 215.0×. It is split into 105 contigs with an N_50_ of 145,273 bp. There are 7,186 predicted genes of which 6,992 are protein coding. The taxonomy of strain FUR100 was determined using the TYGS webserver (v389), closest match being *Rhodococcus erythropolis* JCM 3201^T^ (dDDH d_4_ 88.5%, confidence interval 86.0%–90.5%) (12, 13).

## Data Availability

The draft genome sequence of *Rhodococcus erythropolis* FUR100 has been deposited in GenBank under BioProject accession PRJNA680546, BioSample accession number SAMN16882220, assembly accession GCA_016019085.1, and SRA accession SRR26192813.

## References

[1] Hoydonckx HE, van Rhijn WM, van Rhijn W, de Vos DE, Jacobs PA. 2000. Furfural and derivatives, p 733. In Ullmann’s encyclopedia of industrial chemistry. Vol. 3. Wiley.

[2] Chen LJ, Hecht SS, Peterson LA. 1995. Identification of cis-2-butene-1,4-dial as a microsomal metabolite of furan. Chem Res Toxicol 8:903–906. doi:10.1021/tx00049a001 8555403

[3] Guengerich FP. 2003. Cytochrome P450 oxidations in the generation of reactive electrophiles: epoxidation and related reactions. Arch Biochem Biophys 409:59–71. doi:10.1016/s0003-9861(02)00415-0 12464245

[4] Peterson LA. 2013. Reactive metabolites in the biotransformation of molecules containing a furan ring. Chem Res Toxicol 26:6–25. doi:10.1021/tx3003824 23061605 PMC3574180

[5] Wierckx N, Koopman F, Ruijssenaars HJ, de Winde JH. 2011. Microbial degradation of furanic compounds: biochemistry, genetics, and impact. Appl Microbiol Biotechnol 92:1095–1105. doi:10.1007/s00253-011-3632-5 22031465 PMC3223595

[6] Schmid A, Rothe B, Altenbuchner J, Ludwig W, Engesser K-H. 1997. Characterization of three distinct extradiol dioxygenases involved in mineralization of dibenzofuran by Terrabacter sp. strain DPO360. J Bacteriol 179:53–62. doi:10.1128/jb.179.1.53-62.1997 8981980 PMC178661

[7] Armengaud J, Happe B, Timmis KN. 1998. Genetic analysis of dioxin dioxygenase of Sphingomonas sp. strain RW1: catabolic genes dispersed on the genome. J Bacteriol 180:3954–3966. doi:10.1128/JB.180.15.3954-3966.1998 9683494 PMC107381

[8] Helbich S, Barrantes I, Dos Anjos Borges LG, Pieper DH, Vainshtein Y, Sohn K, Engesser K-H. 2023. The 2-methylpropene degradation pathway in Mycobacteriaceae family strains. Environ Microbiol 25:2163–2181. doi:10.1111/1462-2920.16449 37321960

[9] Jackman SD, Vandervalk BP, Mohamadi H, Chu J, Yeo S, Hammond SA, Jahesh G, Khan H, Coombe L, Warren RL, Birol I. 2017. ABySS 2.0: resource-efficient assembly of large genomes using a bloom filter. Genome Res 27:768–777. doi:10.1101/gr.214346.116 28232478 PMC5411771

[10] Gurevich A, Saveliev V, Vyahhi N, Tesler G. 2013. QUAST: quality assessment tool for genome assemblies. Bioinform 29:1072–1075. doi:10.1093/bioinformatics/btt086 PMC3624806 23422339

[11] Tatusova T, DiCuccio M, Badretdin A, Chetvernin V, Nawrocki EP, Zaslavsky L, Lomsadze A, Pruitt KD, Borodovsky M, Ostell J. 2016. NCBI prokaryotic genome annotation pipeline. Nucleic Acids Res 44:6614–6624. doi:10.1093/nar/gkw569 27342282 PMC5001611

[12] Meier-Kolthoff JP, Göker M. 2019. TYGS is an automated high-throughput platform for state-of-the-art genome-based taxonomy. Nat Commun 10:2182. doi:10.1038/s41467-019-10210-3 31097708 PMC6522516

[13] Meier-Kolthoff JP, Carbasse JS, Peinado-Olarte RL, Göker M. 2022. TYGS and LPSN: a database tandem for fast and reliable genome-based classification and nomenclature of prokaryotes. Nucleic Acids Res 50:D801–D807. doi:10.1093/nar/gkab902 34634793 PMC8728197

